# From Gut to Gain: The Microbiome’s Contribution to Broiler Health and Productivity

**DOI:** 10.3390/vetsci13070633

**Published:** 2026-06-29

**Authors:** Nourhan Nassar, Mohamed Tharwat, Aya Tayel, Muhammad Tariq, Yasir Muhammad Khan, Fahad A. Alshanbari, Ibrar Muhammad Khan

**Affiliations:** 1 College of Life Science, Anhui Agricultural University, Hefei 230036, China; nourhanadel3434@gmail.com; 2 Department of Clinical Pathology, Faculty of Veterinary Medicine, Benha University, Moshtohor 13736, Egypt; 3 Department of Clinical Sciences, College of Veterinary Medicine, Qassim University, P.O. Box 6622, Buraidah 51452, Saudi Arabia; atieh@qu.edu.sa; 4 Department of Food Hygiene and Control, Faculty of Veterinary Medicine, Benha University, Moshtohor 13736, Egypt; aya.magdy@fvtm.bu.edu.eg; 5 College of Animal Science and Technology, Nanjing Agricultural University, Nanjing 210095, China; tariq@stu.njau.edu.cn; 6 Department of Zoology, University of Science and Technology, Banuu 28100, KP, Pakistan; yasirkhan12@gmail.com; 7 Department of Medical Biosciences, College of Veterinary Medicine, Qassim University, P.O. Box 6622, Buraidah 51452, Saudi Arabia

**Keywords:** broiler, gut microbiota, microbial metabolites, antibiotic alternatives, microbiome-host-environment axis, sustainable poultry production

## Abstract

The gut microbiome plays a central role in regulating nutrient utilization, immune function, and disease resistance, thereby directly influencing growth performance and feed efficiency. Existing microbiome modulation strategies, including probiotics, prebiotics, dietary interventions, and antibiotic alternatives, are critically evaluated. Despite their reported benefits, the effectiveness of these approaches often remains inconsistent across production systems. Evidence suggests that this variability is largely driven by complex interactions among microbial communities, host factors, and environmental and management conditions, which are frequently overlooked in conventional intervention-based approaches. To address this gap, this review proposes an integrated microbiome–host–environment framework that links microbial ecology with host physiology and production conditions. The framework provides a systems-level perspective for understanding the factors governing microbiome stability and production responses, offering a basis for more targeted and reliable microbiome management strategies. Finally, current challenges and future research priorities are discussed, including the integration of multi-omics technologies, precision nutrition, and data-driven approaches to support next-generation poultry production systems. By emphasizing the interconnected nature of microbiome regulation, this review contributes a conceptual foundation for improving broiler productivity and sustainability through more consistent and effective microbiome optimization.

## Simple Summary

Improving broiler productivity while reducing the use of antibiotics is one of the major challenges facing modern poultry production. The community of microorganisms living in the chicken gut, known as the gut microbiome, has a profound influence on digestion, nutrient utilization, immunity, and resistance to disease. Although many nutritional strategies have been developed to modify the gut microbiome, their effects are often inconsistent because they are influenced by the bird, its diet, and the surrounding production environment. This review brings together current knowledge on these interacting factors and presents an integrated framework that explains how the microbiome, host, and environment work together to influence broiler performance. It also discusses emerging technologies, including multi-omics, artificial intelligence, precision nutrition, and next-generation microbiome-based interventions that have the potential to enable more precise, effective, and sustainable microbiome management strategies for commercial poultry production.

## 1. Introduction

The global population is growing rapidly and is projected to increase by approximately 2 billion people, reaching 9.7 billion by 2050. In 2020, poultry meat accounted for nearly 40% of global meat production, and its production has increased substantially over the past 30 years [1]. This growth in the poultry industry is due to an increasing human population, economic development and urbanization. Poultry is a major contributor to food and nutrition security, supplying energy, protein and micro-nutrients to humans, with a short production cycle, requiring a large variety of agri-food by-products and waste as inputs to produce human-edible meat and eggs [2]. According to the recent FAO/FAOSTAT statistics, poultry production remains one of the fastest-growing livestock sectors worldwide, driven by increasing demand for affordable animal protein, efficient feed conversion, and relatively low environmental impacts compared with other livestock species [3]. Global poultry meat production has continued to increase during 2023–2025, highlighting the growing importance of optimizing bird health, productivity, and sustainability through improved nutritional and microbiome-based management strategies. The Food and Agriculture Organization of the United Nations (FAO) reported a huge increase in chicken meat production over the past decades. Broilers, specifically bred for rapid growth and efficient feed-to-meat conversion, have revolutionized the poultry industry, enabling large-scale, cost-effective meat production [4,5].

The gut microbiota is closely integrated with host physiology and plays a crucial role in maintaining health and productivity. Under normal conditions, these microbial communities support the host through several key functions. They act as a defensive barrier against pathogenic organisms, help preserve the integrity of the intestinal mucosal lining, contribute to the digestion and metabolism of nutrients, and regulate immune responses. Through these combined actions, the gut microbiota promotes proper growth and development while also protecting the host from harmful pathogens and toxic compounds [6,7]. The digestive process is strongly linked to gut microbiota; nutrient absorption, feed digestibility, energy harvest and therefore productivity are influenced by microbiota composition and diversity [8,9]. The chicken gut microbiota includes hundreds of bacterial species dominated at the phylum level by Firmicutes, Bacteroidetes, Proteobacteria and Actinobacteria [10]. Microbial communities vary along the intestinal tract of chickens, with distinct populations found in different segments such as the crop, gizzard, ileum, cecum, and colon of broiler chickens [11,12]. The gastrointestinal tract of broilers hosts a diverse microbial community that aids in the fermentation of non-digestible carbohydrates, synthesizes essential vitamins, and influences the host’s metabolic processes [13].

Dysbiosis, an imbalance in the gut microbial composition, has been associated with impaired growth, intestinal disorders, and increased susceptibility to infections such as necrotic enteritis, a major concern in commercial poultry production [14]. These changes often result in poor feed efficiency, slower growth rates, greater susceptibility to enteric pathogens, and increased mortality, leading to substantial economic losses for the poultry industry [15]. One of the most important diseases associated with gut microbiota imbalance is necrotic enteritis, a multifactorial intestinal disorder primarily caused by the overgrowth of Clostridium perfringens. Necrotic enteritis is responsible for significant production losses worldwide. The incidence of this disease has become a major concern, particularly following restrictions on the use of antibiotic growth promoters, highlighting the critical role of maintaining a balanced gut microbiota in modern poultry production [16]. Factors such as diet (including fiber, prebiotics, and probiotics), antimicrobial use, and environmental stressors can significantly alter the gut microbiota composition, with direct implications for broiler health and productivity [17]. Beneficial microbes play an important role in breaking down complex carbohydrates, fibers, and proteins, leading to the production of short-chain fatty acids (SCFAs) such as acetate and butyrate. These metabolites support gut health and improve energy utilization, ultimately enhancing feed efficiency and promoting better growth performance [18]. The microbiome also influences host metabolism by synthesizing essential vitamins and amino acids, contributing to muscle growth and overall performance. Additionally, a balanced microbiota supports immune function by maintaining intestinal barrier integrity and reducing inflammation, thereby preventing energy loss to infections [19].

With the ubiquitous need to produce poultry in a sustainable manner in the absence of antibiotics, there is a growing interest in strategies that can modulate the gut microbiome. Probiotics, prebiotics, dietary approaches and other alternatives to antibiotics have shown great promise to improve gut health and production efficiency. New data now indicate that these discrepancies are brought about by multifaceted interactions between microbial communities, host physiology, host genetics, nutrients and environment, which are often studied independently in existing research. Therefore, this review does not focus on the effects of the individual microbiome interventions, but instead suggests a framework of microbiome–host–environment to better elucidate the mechanisms associated with production response variations. This review offers a conceptual basis for more efficient solutions related to microbiome management and creates a new production system for poultry with precision, sustainability, and without the use of antibiotics.

## 2. Data Collection and Selection Criteria

This review was conducted using a structured narrative approach to synthesize current knowledge on the gut microbiome–host–environment axis in broiler chickens. Relevant literature was identified through searches of major scientific databases, including PubMed, Scopus, ScienceDirect, Web of Science, Google Scholar, and Research Gate. Search terms included combinations of keywords such as “broiler gut microbiome,” “gut microbiota,” “microbial metabolites,” “microbiome–host–environment axis,” “antibiotic alternatives,” “probiotics,” “prebiotics,” “synbiotics,” “gut dysbiosis,” “host genetics,” “feed efficiency,” “immune modulation,” and “sustainable poultry production.” Peer-reviewed articles published in English up to 2026 were considered. Priority was given to studies conducted in broiler chickens, particularly controlled experimental trials, field studies, and commercial production investigations that evaluated microbiome composition, function, health outcomes, or production performance. Additional studies involving other poultry species, including turkeys, ducks, and quail, were included when they provided relevant insights into avian microbiome biology. Studies conducted in non-avian species, including pigs, mice, and humans, were not a primary focus of the review. However, such studies were considered when discussing fundamental microbiome mechanisms, host–microbe interactions, or molecular signaling pathways for which poultry-specific evidence remains limited. In these cases, evidence derived from non-poultry models should be interpreted as supportive rather than directly transferable to broiler production systems. The review excluded non-peer-reviewed publications, conference abstracts lacking full data, non-English publications, and studies that did not address microbiome-related outcomes relevant to poultry health, nutrition, immunity, or production performance. Preference was given to recent literature and studies providing mechanistic or applied insights relevant to sustainable and antibiotic-free broiler production.

## 3. Gut Microbiome in Broilers: Composition and Core Functions

### 3.1. Microbial Composition

The chicken gastrointestinal tract harbors a dynamic and complex microbial community composed of bacteria, archaea, fungi, viruses, and other microorganisms. Commonly reported bacterial genera include *Lactobacillus*, *Bacteroides*, *Bifidobacterium*, *Clostridium*, *Faecalibacterium*, and *Ruminococcus*, although their relative abundances differ across studies [20,21]. Microbial composition varies considerably among different segments of the gastrointestinal tract due to differences in pH, oxygen availability, nutrient supply, and transit time. The broiler gastrointestinal tract is functionally compartmentalized, resulting in marked differences in microbial composition and activity among gut segments, as shown in Table 1.

The crop is dominated by *Lactobacillus* species that ferment soluble carbohydrates and contribute to pathogen exclusion [22]. The small intestine, particularly the duodenum, jejunum, and ileum, is primarily responsible for nutrient digestion and absorption and therefore contains microbial communities adapted to rapid nutrient turnover [23]. The ileum generally exhibits lower microbial diversity than the cecum and is commonly enriched with *Lactobacillus*, *Enterococcus*, and another facultative anaerobic bacterium involved in nutrient metabolism. In contrast, the ceca harbor the most diverse and metabolically active microbial ecosystem, functioning as the principal site of anaerobic fermentation, fiber degradation, vitamin synthesis, and SCFA production [24]. Cecal microorganisms are responsible for the breakdown of complex carbohydrates and non-starch polysaccharides, producing SCFAs that contribute to host nutrition and intestinal health. The colon plays a comparatively minor role and is mainly involved in water reabsorption and final microbial processing of digesta [25]. These regional differences are important when interpreting microbiome studies, as microbial functions and production outcomes are highly dependent on sampling location.

Microbial diversity and population density generally increase toward the distal intestinal tract, with the cecum representing the most complex microbial ecosystem. However, the relative abundance of specific bacterial taxa varies according to age, diet, environment, production system, intestinal sampling site, and analytical methodology. Most gut microbial populations are predominantly grouped into two major phyla; the Gram-positive Firmicutes and the Gram-negative Bacteroidetes. The high abundance and diversity of these groups underscore their vital roles in digestion and overall gut health. A well-balanced microbial community, particularly within the cecum, is widely recognized as essential for maintaining optimal gut function [23]. In addition to these dominant phyla, smaller proportions of microbes belong to Actinobacteria (Gram-positive) and Proteobacteria (Gram-negative). In adult hens, Firmicutes and Bacteroidota generally constitute the majority of the adult chicken gut microbiota, whereas Actinobacteriota and Proteobacteria are present in comparatively lower proportions [26]. Nevertheless, these proportions may vary substantially among flocks and experimental conditions, highlighting the importance of considering host, environmental, and methodological factors when characterizing gut microbiota composition.

**Table 1:** Segment-Specific Gut Microbiota and Their Relevance to Broiler Performance.

Gut Segment	Dominant Microbial Groups	Major Functions	Representative Metabolites	Relevance to Broiler Production	References
Crop	*Lactobacillus*, *Enterococcus*	Initial microbial colonization; fermentation of soluble carbohydrates; pathogen exclusion	Lactic acid, acetate	Supports early microbial establishment and reduces pathogen colonization	[27]
Gizzard	Acid-tolerant *Lactobacillus* spp.; relatively low microbial abundance	Mechanical grinding of feed; acidification of digesta; regulation of microbial passage	Organic acids	Improves feed particle breakdown and nutrient accessibility	[22]
Duodenum/Jejunum	*Lactobacillus*, *Enterococcus*, *Streptococcus*	Enzymatic digestion and nutrient absorption; interaction with mucosal immunity	Lactate, amino acid metabolites	Directly affects nutrient digestibility and feed conversion efficiency	[28]
Ileum	*Lactobacillus*, *Clostridium*, *Enterococcus*	Final nutrient absorption; immune regulation; limited microbial fermentation	Lactate, bile acid metabolites	Influences nutrient utilization, intestinal integrity, and immune function	[29]
Ceca	*Bacteroides*, *Ruminococcus*, *Faecalibacterium*, members of *Lachnospiraceae* and *Ruminococcaceae*	Major site of anaerobic fermentation; degradation of non-digestible carbohydrates; SCFA production; vitamin synthesis	Acetate, propionate, butyrate, vitamins B and K	Central to SCFA-mediated immune regulation, gut health, feed efficiency, and growth performance	[30]
Colon/Rectum	Mixed microbial populations originating from the ceca and ileum	Water reabsorption; final microbial processing of digesta	Residual SCFAs	Influences nutrient recovery and litter quality	[30]

### 3.2. Microbiome-Mediated Metabolism, Immunity, and Disease Resistance

#### 3.2.1. Intestinal Barrier Function

Commensal bacteria generate SCFAs and lactate within the gastrointestinal tract, thereby enhancing barrier function and pathogen resistance [31]. For example, xylanase genes have recently been identified and overproduced from the cecum of chickens, facilitating the breakdown and digestion of complex substrates such as non-starch polysaccharides [32]. In addition to these multifunctional functions, the gut microbiota inhibits the colonization of the intestinal tract by pathogens and other non-native microorganisms by competitive exclusion [33,34]. The adhesion of non-pathogenic bacteria to the brush border of intestinal cells inhibits the attachment and invasion of infections. Also, the indigenous gut microbiota inhibits pathogen development by secreting organic acids and bacteriocins, directly stimulating the immune system, and competing for nutrients and adhesion sites on the mucosal membrane [35]. *Lactobacillus* that produces the bacteriocin, Reuterin, was found to be beneficial in preventing the development of *Salmonella*, *Shigella*, *Clostridium*, and Listeria in an in vitro experiment conducted [36]. Increasing these beneficial bacteria, as well as the substrates for their growth and metabolism, improves the host’s feed intake and nutrient utilization. The development of the intestinal host defenses, such as the mucus layer, epithelial layer, and lamina propria, as well as the maintenance of host physiological homeostasis, are all influenced by commensal bacteria, according to experiments comparing conventionally raised with germ-free animals [37].

#### 3.2.2. Immune Development and Regulation

Immune modulation; the intestinal flora is helpful in the training of the immune system to differentiate between normal and pathogenic bacteria, which affects both local and systemic immunity and improves the way a host reacts to pathogens [38]. Consequently, microbiota specifically utilizes IgA and IgG production, which alone may be a reason for several hundred grams of protein during a lifetime, not contributing to the growth of chickens. Another study asserted that IgA targets specifically established gut flora and regulates its abundance by managing species introduced by diet and the environment [39]. In chickens, a considerable amount of total energy expenditure is mainly a result of gut metabolism, predominantly associated with the cellular turnover produced by bacteria [40]. The gut microbiota enhances disease resistance by limiting pathogen colonization through competitive exclusion, competition for ecological niches, and the production of antimicrobial compounds. This protective function is closely linked to microbial metabolic activity, as the gut microbiota provides the host with nutritional molecules via fermentation by-products and other secreted compounds, enzymes, amino acids, and vitamins such as vitamin B and vitamin K [41].

### 3.3. Gut Dysbiosis: Causes and Consequences

Gut dysbiosis refers to any disturbance in the balance, composition, or function of the gastrointestinal microbiota [42]. Moreover, intestinal inflammation may function as both a driver and a consequence of dysbiosis, depending on the physiological context. Factors influencing the equilibrium and progression of the gut microbial ecology can be categorized as external (environmental or management- related) and internal (host-related) [22]. The major causes of gut dysbiosis include overgrowth of pathogens, nutritional imbalance, mycotoxin, antibiotic therapy, improper management, removal of feed supplements, vaccines, infections, stressing factors, harmful chemicals, and genetic predisposition [43]. Dietary factors play a central role in dysbiosis development. Excessive dietary protein or poorly digestible protein sources can induce intestinal inflammation and disrupt gut flora, resulting in dysbiosis. Moreover, intestinal inflammation may function as both a driver and a consequence of dysbiosis. Inadequate digestion and absorption of feed protein provide a substrate for gut microbiota, facilitating the growth of proinflammatory bacterial populations [44]. Mycotoxins produced by fungi represent one of the major anti-nutritional factors in poultry diets and are strongly associated with dysbiosis [45]. Mycotoxins represent one of the most prominent anti-nutritional agents in chicken diets, presenting considerable obstacles to avian health and performance [46]. The increasing interest in implementing the One Health concept for non-antibiotic alternatives seeks to restore and sustain gut health in animal production, consequently markedly impacting the health and wellbeing of broiler chickens [47].

## 4. Microbiome and Broiler Performance

### 4.1. Role of the Intestinal Microbiome in Modulating Performance

The intestinal microbiome is now widely recognized in broiler chicken as a key biological factor influencing growth performance and feed efficiency [48,49]. Increasing evidence shows that both the composition and functional activity of gut microbes are closely linked to important production traits, including body weight gain (BWG), average daily gain (ADG), and feed conversion ratio (FCR) [50]. Recent integrative studies using host genomics and microbiome profiling have shown that gut microbial communities contribute to variation in growth performance and feed efficiency in broilers. Although host genetics may influence microbiome composition, the magnitude of this effect varies across studies and production conditions. Therefore, the gut microbiome is increasingly recognized as a modifiable factor that may contribute to broiler productivity [51]. However, the practical impact of microbiome-associated improvements in traits such as FCR and BWG may vary depending on diet, management practices, environmental conditions, and the consistency of microbiome-targeted interventions. The microbiome is associated with improving nutrient digestibility and intestinal absorption, which is mechanistically how the microbiome increases feed efficiency. Microbial enzymes help to break down otherwise indigestible feed substances, such as non-starch polysaccharides (NSPs), proteins, and lipids. This enzyme activity enhances the availability of metabolizable energy and decreases nutrient losses [52]. Taken together, these results suggest the gut microbiome is a metabolic organ that improves the efficiency of feed utilization and growth performance in a complex of interrelated pathways, such as nutrient digestion, intestinal development, and immune regulation.

### 4.2. Immunomodulatory, Antioxidant, and Anti-Inflammatory Functions of SCFAs

**Figure 1:**
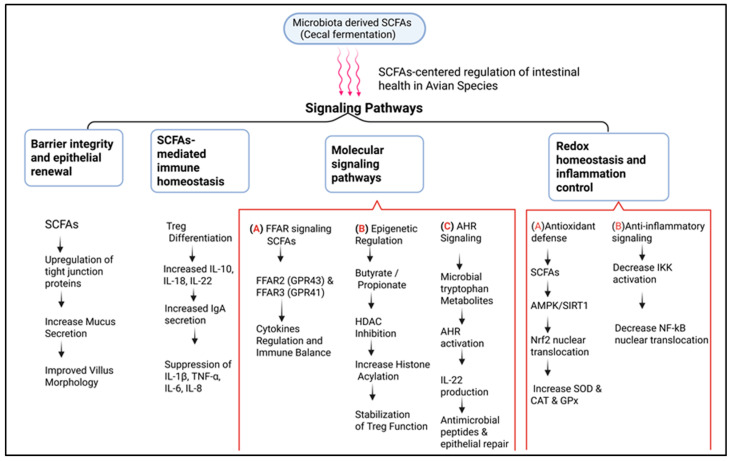
Schematic overview of SCFAs-mediated regulation of intestinal health in avian species. SCFAs produced by cecal microbial fermentation enhance barrier integrity and epithelial function by improving tight junction expression, mucus secretion, and villus morphology. They also maintain immune homeostasis by promoting regulatory T cell differentiation, increasing anti-inflammatory cytokines and IgA production, and suppressing pro-inflammatory responses. At the molecular level, SCFAs act through FFAR2/FFAR3 signaling, epigenetic regulation via HDAC inhibition, and AHR activation. Additionally, they regulate redox balance and inflammation by activating the AMPK/SIRT1–Nrf2 pathway and inhibiting NF-κB signaling.

SCFAs not only serve as an energy source for host epithelium but also as bioactive metabolites that shape intestinal physiology by promoting renewal of epithelium, strengthening the barrier integrity, and modulating mucosal immune response [53,54]. As a result, alterations in the composition of the cecal microbiota and fermentative activity can have a direct effect on the availability of SCFA and the underlying host signaling because SCFA production is a key point of interface between diet, microbiota, and host health [55], as shown in Figure 1. SCFA regulation of gut immunity is primarily based on immune cell differentiation and recruitment and immune factor down-regulation. SCFAs are significant in preventing inflammation of the gut in that they have the capability of transforming T cells into Tregs [56]. Butyrate regulation is attributed to the differentiation and metabolism of macrophages and an increase in antibacterial peptide genes expression [57]. Butyrate contributes to the regulation of IgA synthesis and helps in the elevated IL-10 expression [58]. In broilers, a previous study showed supplementation with *Clostridium butyricum* or sodium butyrate led to upregulation of tight junction proteins like ZO-1, Occludin, and Claudin-1, as well as improved the villus structure of the intestine [59]. The mechanisms of action of SCFAs on intestinal immunity are poorly understood because of the complex relationship with different signaling molecules. There are two major host health/disease control mechanisms with SCFAs. One of them is the regulation of the epigenetics of the target cell, when SCFAs are introduced into the cells, e.g., the repression of histone deacetylase (HDACs) with SCFAs that control the expression of these genes [60]. SCFAs are signaling molecules binding the G-protein-coupled receptors (GPRs; also called FFARs) and it is later applied in the control of various host physiology [61]. In addition, GPRs differ in their tissue distribution and responsiveness to SCFAs. Propionate is a potent agonist of GPR43 (FFAR2), which is primarily expressed in immune cells, enteroendocrine cells, and adipocytes. GPR109A, which is highly expressed in adipocytes, immune cells, and the colon, can also be activated by butyrate and mediates several of its biological effects [62,63]. The impact of SCFAs in immunoregulation through GPRs activation and cytokine regulation has been confirmed in many studies. One study has discovered that GPR41 stimulation by SCFAs enhanced the production of IL-22 that defends against inflammation and promotes gut homeostasis [38]. In chickens, SCFA-related signaling is still under investigation, but similar immune modulation has been suggested. In addition to demonstrating the significance of SCFAs in activating GPR43 on the production of microbiota antigen-specific Th1 cell IL-10, another researcher found that Gpr43−/− mice exhibited more severe intestinal inflammation induced by Dextran Sulfate Sodium than wild-type mice [64].

The antioxidant function of SCFAs is associated with their ability to inhibit the production of oxygen free radicals and mitigate oxidative stress [65,66]. When ROS accumulate beyond the cell’s capacity to neutralize them, oxidative stress can occur, leading to damage of critical cellular components such as proteins, lipid membranes, and DNA. This imbalance has been associated with a range of pathological conditions in livestock and poultry, including intestinal inflammation, impaired digestive function, weakened immune responses, reproductive abnormalities, as well as muscle development disorders, and growth delays [67,68]. Oxidative stress is fundamentally characterized by a disruption of the equilibrium between the generation of free radicals and the organism’s ability to eliminate them, resulting in excessive oxidative burden. To counteract the detrimental effects of ROS, living organisms have evolved a sophisticated antioxidant defense network organized across multiple levels.

The anti-inflammatory pathways of SCFAs, NF-κB regulates gene expression through conventional and noncanonical pathways [69]. Inflammatory and immunological signals activate the classical pathway of NF-κB, utilizing the IKK complex (comprising IKKγ, IKKα, and IKKβ) to phosphorylate IκB, thereby liberating NF-κB dimers that regulate the transcription of proinflammatory cytokines, such as IL-1β and TNF-α [70,71]. The NF-κB pathway is an important regulator of intestinal inflammation and activation of the immune system. Much evidence proves that metabolites of gut microbiota such as SCFAs lead to suppressed activation of the NF-κB pathway in direct or indirect ways through modulation of upstream signaling pathways [72,73]. Also, PRRs such as Toll-like receptors (TLR5 and TLR2) act as the gut’s early warning sensors. They pick up specific signals from bacteria and immediately alert the immune system, triggering the NF-κB pathway to start an inflammatory response when required [74,75]. The regulation of these pathways helps to decrease the inflammatory signaling and enhance intestinal immune homeostasis. Therefore, regulation of NF-κB, TLR-dependent signaling and inflammasome activation is a major way by which SCFA-mediated microbiota remodeling can reduce intestinal inflammation [76,77].

Besides SCFAs, there is a large number of other microbial metabolites that contribute to the improvement of broiler performance. These consist of organic acids like lactate that help in reducing intestinal pH and preventing the proliferation of the pathogenic organisms, and microbial enzymes that aid digestion of complex carbohydrates and proteins, hence the availability of nutrients [37,78]. Moreover, intestinal microbiota produces the necessary vitamins, such as B-complex vitamins and vitamin K, sustaining an important metabolic activity in the host [79]. Notably, these microbial metabolites not only improve nutrient use and growth performance, but also help in disease resistance [80,81].

### 4.3. Sources of Variability and Standardization Challenges in Poultry Microbiome Research

Although the relationship between the gut microbiome and broiler performance is well-established, its outcomes across studies are extremely inconsistent. This inconsistency reflects the complex and multifactorial nature of microbiome-host interactions. This variability in microbiome-mediated responses across studies is driven by multiple interacting host, dietary, environmental, and methodological factors, which collectively determine microbial structure, function, and production outcomes (Table 2). Host genetics is one of the most important sources of variability that affects the microbial composition and metabolic responses [82]. Recent studies that combine genomic and microbiome data have shown that the genotype-microbial community interaction has a major impact on feed efficiency and growth phenotype, implying that microbiome effects cannot be universal and universally applicable [83]. Another key factor that contributes to the variability is diet composition. Feed ingredient (e.g., corn- vs. wheat-based feeds), fiber, and nutrient composition changes in microbial substrates and fermentation patterns, resulting in different microbiome structure and functional products [84]. Minor modifications in diet formulation may lead to major changes in microbial structure and the production of metabolites. Microbial colonization and stability can be affected by housing conditions, stocking density, hygiene, stress and antibiotic use [85]. Also, variation in microbiome modulation strategies- such as the choice of probiotic strain, dose and time shows inconsistencies in the results of the studies. Not every probiotic strain has the same effect and the effect is conditional on the compatibility of the probiotic strain with the host microbiome and the environment [86,87].

Lastly, microbiome research is subject to substantial variability due to methodological differences. The microbiome composition and function of broilers is dynamic and varies greatly during their development. Age at sampling can have a significant effect on the results obtained [88]. Similarly, the location of the sampling (crop, ileum, cecum or fecal samples) can yield significantly different microbial profiles, due to different microbial communities in each section of the gut [89]. There is also some variation in how samples are handled, in the method used to extract DNA from the samples, in the type of DNA sequencing platform used, in the region of the 16S rRNA gene chosen for sequencing, in the depth of the sequencing process, and in the bioinformatics pipelines used for data processing and taxonomic classification [90]. In addition, the use of taxonomic profiling alone might miss certain functional differences between microbial communities. The expansion of functional metagenomics and other multi-omics techniques has shown that microbial function may play a more important role than taxonomic composition in explaining production outcomes, especially when these are increased.

There is a huge methodological variability among poultry microbiome studies, which calls for some standardization. Harmonized protocols for sample collection, preservation, DNA extraction, sequencing, and data analysis would be helpful for future research. But as important is a full description of the metadata, such as detailed genetic information on the host, diet formulation, environmental conditions, housing systems, management practices, health status and antibiotic exposure. Such standardized reporting of dietary composition and environmental variables would help ensure the reproducibility of studies and allow for meaningful comparisons [90]. This model can be used to develop standardized experimental designs, integrative multi-omics analyses and precision nutrition approaches to enhance poultry productivity.

**Table 2:** Context-dependent drivers of variability in broiler gut microbiome and production responses.

Factor Category	Specific Variable	Effect on Microbiome	Mechanistic Basis	Outcomes/Production Impact	Supporting Evidence
Host genetics	Breed, genetic line, growth selection	Modulates microbial community structure	Host genetics influences gut physiological environment, affecting microbial colonization	Genetic variation leads to differences in host–microbe interactions and microbial recruitment leas to variation in FCR, BWG, and disease resilience among broiler lines	[91,92,93,94]
Diet composition	Fiber type, protein level, fat source	Modulates microbial diversity, abundance of beneficial taxa, and fermentation profiles	Microbial metabolism is substrate-dependent: NSPs are fermented to SCFAs, whereas excess protein promotes proteolytic fermentation and potentially harmful metabolites	Diet formulation differences result in variable microbial and metabolic responses	[95,96,97,98]
Environment & housing	Litter quality, ammonia level, ventilation, farm conditions	Induces shifts in gut microbial composition and may reduce microbial stability (dysbiosis)	Environmental exposure to ammonia and litter conditions alter microbial balance and pathogen load	Variability in housing management (e.g., litter reuse, ventilation rate, ammonia concentration) leads to inconsistent microbial exposure and colonization patterns	[99,100,101,102]
Age/developmental stage	Early vs. mature microbiome	Dynamic succession and increasing diversity	Initial colonization by facultative aerobes followed by replacement with obligate anaerobes, leading to a more stable and diverse microbial community	Early life microbiome is highly unstable and sensitive to environmental and dietary interventions	[103,104,105]
Feed management practices	Feed form (pellet vs. mash), feeding strategy	Alters microbial composition, fermentation activity, and spatial distribution along the gut	Alters digesta flow and substrate availability	Differences in feed processing (pelleting, particle size) and feeding systems across farms lead to variable gut conditions	[106,107]
Pathogens & disease pressure	*Salmonella*, *Clostridium perfringens*	Induces dysbiosis characterized by reduced beneficial bacteria (e.g., *Lactobacillus*) and shifts in microbial community structure	Pathogen colonization modifies microbial composition, promotes inflammation, and disrupts competitive exclusion, allowing pathogen persistence and overgrowth	Infection pressure varies with biosecurity, hygiene, flock management, and environmental exposure lead to increase mortality, reduce growth performance, and impaired feed efficiency	[108,109]
Methodological differences	Sampling site, sequencing method	Produces variable microbial profiles across studies	Differences between intestinal regions (e.g., ileum vs. cecum) and sequencing approaches (16S vs. metagenomics) lead to variation in detected taxa and resolution	Lack of standardized sampling protocols and analytical pipelines across studies	[103,110]

## 5. Microbiome-Targeted Strategies for Enhancing Broiler Productivity and Health

### 5.1. Probiotics

**Figure 2:**
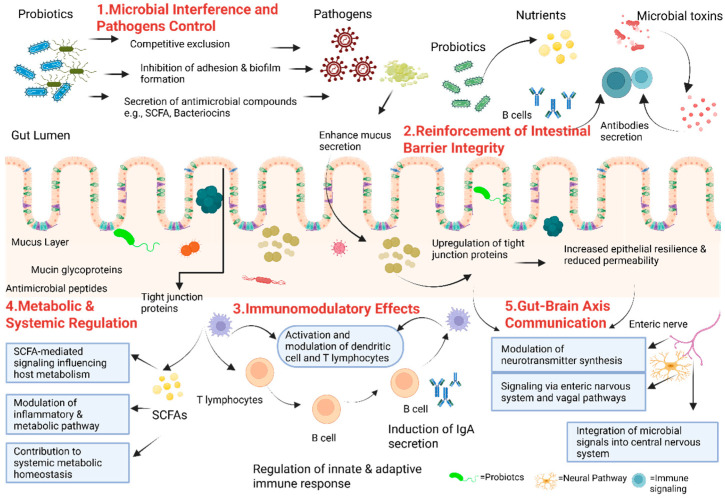
Mechanisms of probiotic action in broiler chickens. Probiotics improve host health and performance through multiple pathways: (1) inhibition of pathogen colonization via competitive exclusion and antimicrobial production; (2) reinforcement of intestinal barrier integrity by enhancing mucus secretion and tight junction expression; (3) immunomodulation through activation and regulation of immune cells; (4) metabolic regulation via production of bioactive metabolites such as SCFAs; and (5) modulation of the gut–brain axis through microbial signaling pathways.

The idea that gut microbes influence host health dates back to the early 20th century, when the future Nobel laureate Elie Metchnikoff first proposed a beneficial role for intestinal microbiota. Interest in this concept was revived decades later by a short report published in Nature in 1973 by Nurmi and Rantala [111], who demonstrated the use of bacterial cultures to control Salmonella infections in poultry. This work helped lay the foundation for the use of direct-fed microbials, or probiotics, in animal production. Since then, numerous studies have shown that probiotics can help restore microbial balance in the gut and introduce beneficial functions within the microbial community, leading to decreased gut inflammation and infections while improving overall performance in poultry [37,112,113]. The principal mechanisms through which probiotics enhance gut health and broiler performance are summarized in Figure 2, including competitive exclusion of pathogens, reinforcement of intestinal barrier integrity, immunomodulation, production of beneficial microbial metabolites, and regulation of gut–brain signaling. Probiotics are live microorganisms and referred to as direct-fed microbials because they have beneficial effects on the host by improving the gut health and nutrient utilization of the animal [114,115,116].

For example, probiotic supplementation has been shown to improve the immune response, structure and cytokine production in farm animals, which has a positive effect on intestinal mucosal barrier against pathogens [117]. Probiotics can be sourced from a variety of origins, including milk, fermented foods, fecal material, and the gastrointestinal tract of different animals [118]. Among these, traditional fermented foods are one of the most common sources, particularly for lactic acid bacteria and *Bifidobacterium* species, although a wider range of probiotic strains is now commercially available [119,120]. The production of enzymes by different bacterial strains has led to the rapid expansion and development of probiotics. *Bacillus licheniformis* strains have been widely used in the industry for their amylase, alkaline, protease, keratinase and B-mannanase production [121]. The feeding of *B. subtilis* strains to poultry has been reported to enhance gut health, IgAs (immunoglobulin A) production in the duodenum and FCR [122]. Selected *Lactobacillus* strains have been reported to enhance intestinal immunity against coccidiosis infection by affecting the intraepithelial lymphocyte population with surface marker cluster of differentiation 4 (CD4) [123]. Probiotics’ enteric immunostimulatory effect could also be due to their capacity to stimulate T lymphocytes in the gut immune system through increasing Toll-like receptor (TLR) activity [124].

### 5.2. Prebiotics and Synbiotics

Dietary fiber (DF) includes a wide range of non-digestible carbohydrates, such as cellulose, hemicellulose, pectins, and non-starch polysaccharides (NSPs) [17,125]. However, not all dietary fibers act as prebiotics. Prebiotics are specific substrates that can be selectively utilized by beneficial gut microorganisms, thereby promoting host health. In addition, the breakdown of NSPs by exogenous enzymes can generate oligosaccharides that may also exhibit prebiotic properties, depending on their structure and susceptibility to microbial fermentation [126]. Several of these fiber-derived oligosaccharides have been reported to promote beneficial gut bacteria, enhance immune function, improve intestinal health, and increase the production of SCFAs in poultry [17]. These approaches help supply fermentable substrates that support the growth and activity of beneficial gut microorganisms [32,127]. It has been observed that DF is fermented by lower gut microbes to form SCFA [128]. But due to their indigestibility, they decrease the apparent metabolizable energy (AME) of feed, and therefore increase the viscosity of digesta, which in turn will decrease the digestibility of other nutrients [129]. Thus, processing and enzyme addition to enhance the digestibility of fiber will also enhance the digestibility of other nutrients in the feed and increase fermentable substrate for the gut microbes. The fermentable substrate could be complex fragments or simply oligomers that could play the role of prebiotics [17,130,131].

The impact of oligosaccharides on the gut microbiota of poultry depends on several factors, including their inclusion level and molecular structure. In general, oligosaccharides with a lower degree of polymerization tend to be fermented more readily by intestinal microorganisms than longer-chain carbohydrates. However, responses are not always consistent across studies, as the effects may vary with the amount supplemented and the characteristics of the basal diet. Therefore, both the dosage and structural properties of prebiotic compounds should be carefully considered when evaluating their potential benefits in poultry nutrition [132]. DF in poultry diet can help cellulolytic and beneficial bacteria (such as *Lactobacillus* and *Bifidobacterium*) and increase the SCFA produced, and the synergism of both avoids digestive disorders and wet litter [133]. The feed ingredients containing fiber with prebiotic potential have the benefit of stimulating such commensal and beneficial microbes that are resident in the GIT of the host [134,135]. Although dietary fiber can provide important benefits for gut health, its effects are not always positive and largely depend on the type and amount included in the diet. For example, high levels of soluble NSPs may increase digesta viscosity, which can interfere with nutrient digestion and absorption and ultimately reduce bird performance. In addition, the effects of fiber and prebiotic supplementation can vary according to the composition of the basal diet, including the cereal source, NSP content, protein level, and the availability of other fermentable substrates. Therefore, the successful use of dietary fiber in poultry nutrition requires careful consideration of both fiber characteristics and overall diet composition [136]. Therefore, the fermentable DF can alter the gut microbiome and enhance the growth of beneficial bacteria which would be needed to enhance the performance of broilers in the absence of AGP. However, further research is required to better understand how different components of dietary fiber interact with gut microbes within the highly competitive intestinal environment.

### 5.3. Antibiotic Alternatives

Antimicrobial resistance (AMR) is a global catastrophic threat to modern medicine and its successes and is a serious health concern [137,138]. There have been a lot of studies seeking to identify natural products with the same positive impact as growth promoters. Among the most widely used are probiotics, prebiotics, exogenous enzymes, organic acids, immunostimulants, bacteriocins, bacteriophages, phytogenic feed additives, plant-derived compounds (phytocides), nanoparticles, and essential oils [139,140], as shown in Table 3. Plant-derived feed additives are obtained from herbs and other botanical sources and are widely used to support animal production. Their use has increased due to reported beneficial effects on growth performance, immune function, and stress resilience, although responses are variable. Recent studies suggest that these phytogenic additives can serve as effective alternatives to antibiotic growth promoters while also improving growth outcomes in broiler chickens [141,142]. Nevertheless, their effects are not always consistent and are influenced by factors such as plant source, chemical composition, processing methods, dosage, encapsulation techniques, basal diet, and experimental challenge conditions. Therefore, their application should be interpreted cautiously, particularly in terms of reproducibility under commercial production systems. Recent evidence further supports the use of phytogenic compounds and probiotics as sustainable alternatives to antibiotic growth promoters in poultry production. Curcumin, the principal bioactive compound of turmeric (*Curcuma longa*), has attracted considerable attention due to its antimicrobial, antioxidant, anti-inflammatory, and immunomodulatory properties [143]. Consequently, curcumin has been proposed as a promising green antibiotic substitute in modern poultry production systems [144]. Similarly, probiotic supplementation with *Clostridium butyricum* has emerged as an effective strategy for promoting intestinal health in poultry. Recent findings indicate that *C. butyricum* supplementation improves gut microbial balance, intestinal barrier function, and overall health status, highlighting its potential as a viable alternative to antibiotic growth promoters in poultry diets [145].

**Figure 3:**
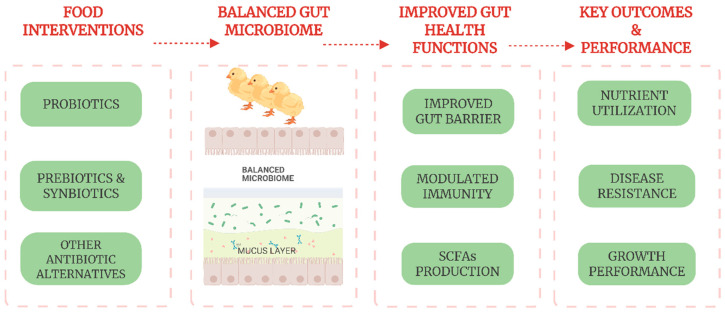
Mechanisms of microbiome modulation strategies in broiler chickens. Probiotics, prebiotics/synbiotics, and antibiotic alternatives modulate the gut microbiome through complementary mechanisms, including promotion of beneficial bacteria, inhibition of pathogens, and production of SCFAs.

Essential oils are the oily liquids of fragrant and volatile aromatic substances of a plant. Essential oils may be derived from plant sources or produced synthetically, although only a limited number exhibit strong antimicrobial activity [135]. Common examples include thymol, trans-cinnamaldehyde, carvacrol, and eugenol. These compounds exert their effects by disrupting bacterial enzyme systems and by modulating immune responses and inflammatory processes [139]. Organic acids are preservatives preventing microbial and fungal invasion in feed. They are mostly carboxylic acids with a hydroxyl group on their alpha carbon like malic, lactic and tartaric acid. Organic acids’ addition to drinking water has a protective effect on young chicks from *Campylobacter* infection [146,147,148]. The poultry gut ecosystem is crucial in the detoxification and removal of pathogens from the poultry gut. The gut microbial population is highly influenced by various factors such as feed additives (phytobiotics, prebiotics, probiotics, feed enzymes, organic acids), feed formulation, genetics, heat stress, and feed management, as shown Figure 3. Dietary addition of bioactive compounds of cinnamon helps in the creation and maintenance of microflora and digestive functions in poultry. They found that dietary cinnamon increased the growth of beneficial bacteria and decreased the pathogenic bacteria load in comparison to the control [139,149]. They observed that the dietary cinnamon enhanced the growth of *Lactobacillus* spp. and inhibited *Campylobacter* spp. and *E. coli* in the ileum and cecum of chickens. The SCFAs production is due to the fermentation of the *Lactobacillus* spp. and is responsible for maintaining the gut environment and inhibiting the pathogenic bacteria that are sensitive to pH. The phytochemicals of cinnamon may have anti-microbial properties against *Enterococcus faecalis*, *Vibrio parahaemolyticus*, *Pseudomonas aeruginosa*, *Salmonella* spp., *Klebsiella pneumoniae*, *Staphylococcus epidermis*, *Staphylococcus aureus* and *E. coli* [150]. Flavanols are abundant in cinnamon, which have high antioxidant and anti-microbial activities. Flavanols, such as quercetin were reported to suppress the growth of bacteria, *Salmonella enterica* serotype *Typhimurium*, *E. coli*, *Staphylococcus aureus*, and *Pseudomonas aeruginosa* in the poultry gut [151,152]. Also, anthocyanins and their metabolites influence the gut by increasing *Lactobacillus* spp., *Enterococcus* spp., *Bifidobacterium* spp. in the poultry gut. Anthocyanins can inhibit the growth of pathogenic bacteria and enhance the organ functions in poultry and can be used as a heat stress mitigating agent [153]. Moreover, these supplements may even improve the gene expression of immune and intestinal barrier function, resulting in improved gut health [154].

**Table 3:** Mechanistic overview of antibiotic alternatives in poultry and their effects on gut microbiota, intestinal integrity, and immune function.

Functional Category	Key Agents	Mechanisms of Action	Molecular Targets and Signaling Pathways	Microbiota Modulation	Gut Barrier Integrity	Immunological Effects	Potential for Synergy	References
Phytogenic Feed Additives	Garlic (allicin); Cinnamon (*C. verum*); Thyme; Oregano extracts; Clove (eugenol); Green tea catechins	Disturbance of bacterial membranes; quorum sensing inhibition; antioxidant activity	Down-regulation of NF-κB; modulation of MAPK pathways; up-regulation of tight junction proteins (ZO-1, occludin, claudins)	Enhancement of *Lactobacillus* and *Bifidobacterium*; suppression of *E. coli*, *Salmonella*, *Campylobacter*	Increased villus height; improved villus height to crypt depth ratio; enhanced mucosal barrier	Improved IgA and IL-10; reduced TNF-α and IL-6	Strong synergy with probiotics, organic acids, and enzymes	[155,156,157,158]
Phytogenic Bioactive Compounds	Capsaicin; Piperine; Curcumin; Cinnamaldehyde; Carvacrol; Thymol	Anti-inflammatory, antioxidant, and antimicrobial activities	Suppression of NF-κB signaling; suppression of COX-2 and iNOS; modulation of oxidative stress pathways	Increased of beneficial microbiota and inhibition of pathogens	Increased goblet cell density and mucin (MUC2) expression	Reduced IL-1β and TNF-α; increased IL-10 and IL-4	Effective when combined with probiotics and organic acids	[159,160]
Essential Oils	Thymol; Carvacrol; Eugenol; Trans-cinnamaldehyde; Citral; Menthol	Interruption of membrane integrity and enzymatic systems	Collapse of proton motive force; inhibition of ATP synthesis	Reduction of pathogenic bacteria; support of lactic acid bacteria	Improved epithelial integrity and tight junction expression	Enhanced innate immunity; reduced physiological stress markers	Enhanced efficacy through encapsulation and nano-delivery systems	[161,162]
Organic Acids	Formic acid; Acetic acid; Propionic acid; Butyric acid; Lactic acid; Citric acid	Acidification of gastrointestinal tract; intracellular pH disruption	Epigenetic modulation via HDAC inhibition; activation of barrier-related genes	Increased populations of *Lactobacillus* and *Bifidobacterium*	Enhanced villus morphology and epithelial barrier function	Increased anti-inflammatory cytokines and immune regulation	Strong synergy with essential oils and probiotics	[163,164]
Probiotics	*Bacillus subtilis*; *B. licheniformis*; *Lactobacillus plantarum; Lactobacillus acidophilus*; *L. plantarum*; *Enterococcus faecium*; *Saccharomyces boulardii*	Competitive exclusion of pathogens; production of antimicrobial peptides and enzymes	Modulation of gut-associated lymphoid tissue and cytokine signaling	Increased microbial diversity and beneficial bacteria	Increased villus height and reduced intestinal permeability	Enhanced IgA and IgY production; reduced inflammatory cytokines	Strong synergy with prebiotics (synbiotic effect)	[165,166,167]
Prebiotics	MOS; FOS; GOS; Inulin; XOS	Selective stimulation of beneficial microbiota; inhibition of pathogen adhesion	Activation of Toll-like receptor pathways and fermentation processes	Enrichment of *Lactobacillus*, *Bifidobacterium*, and *Roseburia*	Improved gut morphology and goblet cell proliferation	Enhanced mucosal immunity and IgA production	Highly synergistic with probiotics	[168,169]
Synbiotics	*Lactobacillus* + FOS; *Bacillus* + MOS; multi-strain probiotics + inulin	Combined probiotic and prebiotic effects; enhanced SCFA production	Upregulation of mucin genes and tight junction proteins	Strong enrichment of beneficial microbiota	Significant improvement in villus architecture and barrier function	Reduced pro-inflammatory cytokines; enhanced immune responses	Represents the most effective synergistic approach	[170]
Bacteriophages	*Salmonella* phages; *E. coli* phages; *Campylobacter* phages	Targeted bacterial lysis via replication within host cells	No direct interaction with host cellular pathways	Specific elimination of pathogens without disrupting commensals	Indirect protection of intestinal integrity	Reduced systemic inflammation and immune stress	Can be combined with probiotics or organic acids	[171]
Immunostimulants	β-glucans; Mannans; Nucleotides; Yeast extracts; Chitosan	Activation of innate immune receptors	Activation of dectin-1, TLR-2, and cytokine signaling pathways	Indirect support of beneficial microbiota	Enhanced mucosal barrier and mucin production	Increased macrophage and natural killer cell activity	Synergistic with vaccines and probiotics	[172]
Flavonoids/Polyphenols	Quercetin; Catechins; Anthocyanins; Resveratrol; Kaempferol	Antioxidant and antimicrobial activities; modulation of signaling pathways	Inhibition of NF-κB; activation of antioxidant enzymes (SOD, CAT, GPx)	Promotion of beneficial microbiota and suppression of pathogens	Improved intestinal morphology and barrier function	Reduced inflammatory cytokines; improved immune balance	Effective when combined with phytogenic additives	[173]
Nanotechnology-Based Additives	Nano-encapsulated phytochemicals; Chitosan nanoparticles; Liposomal essential oils;	Controlled release and enhanced bioavailability	Improved cellular uptake and stability	Preservation of beneficial microbiota while targeting pathogens	Enhanced epithelial barrier integrity	Reduced systemic inflammation	Enhances efficacy of phytogenic and antimicrobial compounds	[174]
Feed Enzymes	Phytase; Protease; Xylanase; β-glucanase; Amylase	Improved nutrient digestibility and reduction of anti-nutritional factors	Release of bound nutrients; modulation of gut environment	Indirect promotion of beneficial microbiota	Improved intestinal morphology through enhanced digestion	Reduced gut inflammation	Strong synergy with probiotics and prebiotics	[175]

Note: The molecular targets and signaling pathways included in this table have been identified in the poultry species as well as from mammalian models in which the evidence for the poultry species is limited. In broiler chickens, where direct evidence is lacking, proposed mechanisms are proposed pathways that need further validation in avian systems.

## 6. Environmental and Management Factors Affecting the Microbiome

### 6.1. Impact of Housing, Lighting, and Stocking Density on the Gut Microbiota

The housing environment is a critical determinant of the gut microbiome in broiler chickens, influencing microbial diversity, pathogen load, and overall gut health. Housing systems vary widely, ranging from conventional intensive systems (high-density, controlled-environment barns) to free-range or organic systems, each exerting distinct effects on the microbial ecology of the gut [176,177]. Stocking density is a major factor; overcrowding increases stress levels, fecal-oral contamination, and ammonia concentrations, which can disrupt the gut microbiota by favoring pathogenic bacteria such as *Clostridium perfringens* and *Escherichia coli* while suppressing beneficial microbes like *Lactobacillus* spp. High ammonia levels, a byproduct of poor ventilation and wet litter, damage the respiratory and intestinal epithelium, compromising gut barrier function and altering microbial composition [178,179].

Litter quality and management also play a pivotal role; wet or caked litter promotes the proliferation of harmful bacteria and fungi, whereas dry, well-maintained litter with proper aeration supports a more balanced microbiome. The type of litter material (e.g., wood shavings, rice hulls, or straw) further influences microbial populations due to differences in moisture retention and microbial load [180]. Ventilation and temperature control are crucial, as poor airflow leads to heat stress and humidity buildup, both of which negatively impact gut microbiota. Heat stress, in particular, reduces gut integrity, increases intestinal permeability, and shifts microbial communities toward pathogenic species [157,181]. Conversely, well-ventilated houses with optimal temperature and humidity levels promote microbial stability and reduce disease incidence. Lighting regimens (e.g., continuous vs. intermittent lighting) also affect gut microbiota by altering feeding patterns and stress responses, which in turn influence microbial metabolism. Additionally, biosecurity measures, such as disinfection protocols and rodent/pest control, prevent the introduction of external pathogens that could disrupt the gut microbiome. Alternative housing systems, such as free-range or pasture-raised setups, generally foster greater microbial diversity due to exposure to soil-based microbes and varied foraging materials, which can enhance immune development and gut resilience [182,183]. However, these systems also pose challenges, including higher exposure to environmental pathogens like *Campylobacter* and *Salmonella*. Modern advancements, such as automated environmental controls (e.g., precision ventilation, heating, and cooling systems), help maintain stable conditions that support a healthy microbiome [184]. The transition to antibiotic-free production has further emphasized the need for optimal housing conditions to prevent dysbiosis and disease outbreaks [185]. Future strategies may include microbiome-friendly housing designs, such as enriched environments with probiotics in litter or water systems, to promote beneficial microbial colonization. Overall, optimizing housing conditions is essential for maintaining a stable and beneficial gut microbiome in broilers, which directly correlates with growth performance, feed efficiency, and disease resistance.

### 6.2. Feed Management

One of the strongest factors that determines the gut microbiome is the feed composition and feeding methods. Substrate availability due to the type, quality, and processing of feed affects microbial fermentation, and thus, microbial composition and metabolic products [186,187]. The components of the diet, including fiber, protein and fat have different roles to play in the modulation of microbes. Non-digestible carbohydrates stimulate the development of the beneficial bacteria and the generation of the SCFAs, and the surplus protein can support the proliferation of proteolytic and, possibly, detrimental bacteria [188]. The feeding methods, such as the form of feed (pellet or mash), the frequency of feeding and the nutrient content in the feed also determine the microbial colonization and activity [189]. Also, there are a plethora of feed additives like probiotics, prebiotics, enzymes, and phytogenics that are commonly used to regulate the microbiome. They are effective, however, depending on several factors such as dosage, strain specificity, and interaction with existing microbial communities. Differences in feed management practices among production systems lead to significant differences in microbiome composition and performance outcome [190].

### 6.3. Stress Factors

Stress is a significant modulator of the gut microbiome and it is capable of severely affecting the performance of broilers. As environmental stressors, heat, overcrowding, transportation, and handling disturb the homeostasis of the gut by altering the microbial composition, and enhancing the intestinal permeability [191]. Heat stress leads to dysbiosis which is commonly marked by loss of microbial diversity and an overabundance of pathogenic microbes [14]. For instance, alpha diversity has been extensively studied across various trials focusing on the impact of heat stress on chicken microbiota. Commonly utilized indices, such as observed species, Chao1, Faith’s phylogenetic diversity, Pielou’s evenness, Shannon, and Simpson indices, were employed to describe species richness and evenness [191,192]. Indeed, other experimental variables with a more substantial impact on gut microbiota may have been the primary cause [192]. For example, factors such as the initial state of the microbiota before heat exposure should not be disregarded. Evidence suggests that microbial communities exhibit varying levels of resilience, depending on their composition. Studies found evidence of significant beta diversity differences between the control and heat stress treatment groups [193]. Notably, this difference in beta diversity was observed even in trials that did not demonstrate differences in alpha diversity or abundance of microbes at higher taxonomic ranks. For instance, beta diversity analyses revealed heat-induced microbial shifts in the cecum of broilers; however, no significant effect on the abundance of Bacteroidetes and Firmicutes was observed. Similarly, Wang et al. [194] found a limited influence of heat stress on the relative abundance of major phyla in the ileum of broilers; however, significant clustering related to ambient temperature was observed. One potential explanation for this phenomenon is that heat stress affects the abundance of microbial taxa to varying degrees, with lower taxonomic ranks, such as species and genera, being more susceptible. It is well established that HS leads to an increased heat load within the internal organs of birds. Shi et al. [195] demonstrated that the cecal microbiota of broilers exhibits stronger heat-induced shifts with increasing age. Indeed, heat stress had little effect on the microbial structure of 15-day-old broilers but resulted in completely distinct clusters in 42-day-old birds. It is well established that older birds are more susceptible to heat stress owing to their body weight and feather coverage, which affect their ability to dissipate heat [196].

High stocking density induces chronic stress, poor air quality, and limited movement in poultry, triggering gut dysbiosis (an imbalance in the microbial community). This ultimately causes a decrease in nutrient absorption, compromised intestinal barrier integrity, and reduced growth performance [179]. Transportation induces significant stress on poultry, triggering a “fight-or-flight” hormonal response (elevated corticosterone) that alters the gut environment. This stress leads to stress-associated dysbiosis, a reduction in beneficial bacteria (such as *Lactobacillus* spp.), and a corresponding increase in potential pathogens (like *E. coli* and *Clostridium* spp.) [197].

## 7. Toward an Integrated Microbiome-Host-Environment Framework

**Figure 4:**
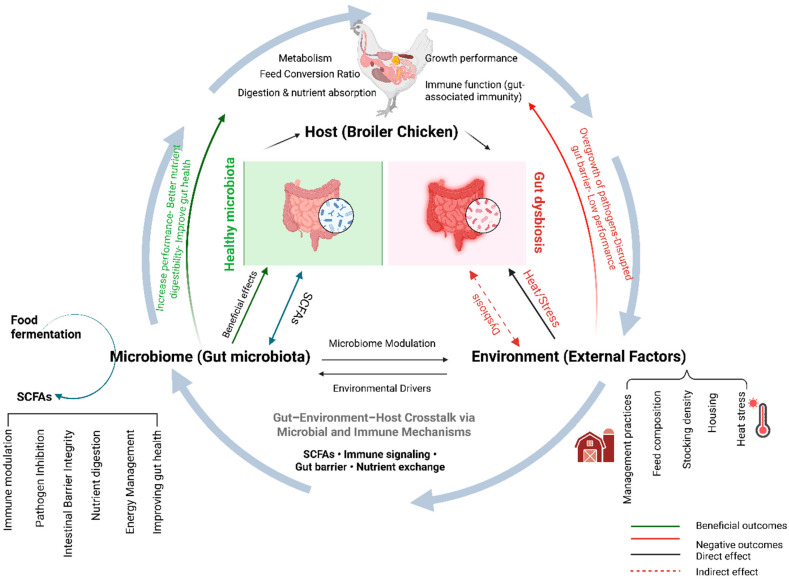
Integrated microbiome–host–environment axis in broiler chickens. This schematic illustrates the dynamic interactions between the gut microbiota, host physiology, and environmental factors in broiler chickens. A balanced microbiome enhances nutrient digestion, SCFA production, gut barrier integrity, and immune function, thereby improving growth performance and FCR. In contrast, environmental stressors such as heat stress, poor management, and suboptimal feed can disrupt microbial balance, leading to gut dysbiosis, pathogen overgrowth, and reduced performance. Bidirectional crosstalk between the microbiome, host, and environment is mediated through microbial metabolites, immune signaling, and nutrient exchange, ultimately influencing health, productivity, and disease resistance.

Current strategies for managing the microbiome in broiler production tend to rely on individual interventions probiotics, dietary changes, or adjustments to the rearing environment without considering how these factors might interact. Yet in practice, the success of such approaches varies widely from one study to the next, and even among commercial systems. A key shortcoming of these existing methods is the implicit assumption that microbiome-targeted strategies work independently of the host animal and its surroundings. In reality, broiler performance is shaped by a tangled web of ongoing interactions among the gut microbiome, the bird’s own physiology, and the production environment [198,199]. The framework we present in this review moves beyond conventional intervention-based models by bringing these three domains together into one coherent conceptual system, as shown in Figure 4.

What makes this framework particularly useful is that it helps explain a long-standing puzzle: why responses to probiotics, prebiotics, phytogenic additives, and similar microbiome-targeted interventions are so often inconsistent. The same product or treatment can yield very different results depending on the setting, simply because its effectiveness is shaped by how microbial ecology, host traits, and environmental factors play out together in a given situation. This means we need to stop thinking about microbiome optimization as a matter of tweaking individual bacterial groups or feed additives in isolation. Instead, it calls for a systems-level approach that recognizes the whole picture. This conceptual framework also establishes a foundation for future precision poultry production. By integrating microbiome profiling, host phenotyping, environmental monitoring, and data-driven decision support systems, it may become possible to develop predictive models capable of identifying optimal intervention strategies for specific production settings. Such an approach would improve the consistency and effectiveness of microbiome-based solutions while supporting sustainable and antibiotic-free poultry production.

From a practical perspective, the proposed framework can serve as a guide for integrating microbiome data with nutritional, environmental, health, and production parameters in broiler systems. Researchers and producers may use this approach to identify microbial signatures associated with desirable outcomes such as improved FCR, growth performance, disease resistance, and animal welfare. Furthermore, the framework can support the development and evaluation of targeted interventions, including dietary modifications, probiotics, prebiotics, and management strategies aimed at optimizing gut health. The framework may also assist in the design of future experimental studies by encouraging the simultaneous assessment of microbiome composition, microbial metabolites, intestinal morphology, immune responses, production performance, mortality, and welfare indicators. Such integrated approaches can improve understanding of causal relationships between the gut microbiome and broiler productivity under commercial production conditions.

## 8. Challenges and Future Perspective

Despite substantial advances in the characterization of the broiler gut microbiome, significant challenges remain in translating microbiome research into practical applications for poultry production. Multiple issues remain to be explored to allow for implementing the findings in commercial production practices. It is essential to identify and validate robust biomarkers of microbial communities reflecting feed efficiency, growth, resistance to diseases, and poultry welfare. Thus, future research should use standardized experimental design and datasets obtained from multiple locations to identify whether some microbial taxa, microbial consortia, or metabolome profiles can serve as reliable predictors of production outcomes in different strains of birds and management regimes.

It is also necessary to switch from merely descriptive taxonomy to functional microbiomics. This involves using such molecular techniques as metagenomics, meta-transcriptomics, metabolomics, and host transcriptomics to identify the microbe-related pathways, metabolites, and microbes themselves that positively affect nutrition, immune regulation, and gut health. Of special interest is the study of the mechanism by which microbiota produce metabolic products influencing the resistance to diseases like necrotic enteritis. Precision poultry farming and precision nutrition are emerging concepts that may contribute to improving poultry production through data-driven optimization of feeding strategies, environmental management, and microbiome modulation.

Future studies should also focus on standardizing sampling and analytical protocols to reduce variability across studies. Longitudinal studies tracking birds from hatch to slaughter are needed to better understand microbiome development over time. In addition, controlled challenge experiments should be conducted to establish causal relationships between microbial changes and health or performance outcomes, rather than relying solely on correlation-based observations. Field validation under commercial production conditions, along with economic evaluations of microbiome-targeted interventions, will be essential to determine their practical feasibility and scalability. Furthermore, future research should integrate microbiome data with host phenotypes, welfare indicators, and sustainability metrics to provide a more comprehensive assessment of production systems.

Artificial intelligence and machine learning can also contribute to solving the problems of precision poultry production. Future research should apply these computational techniques to develop predictive models combining information about the microbiome, nutrition, environment, and production metrics to predict indicators such as feed conversion rate, body weight, or risk of diseases. The models need to be tested in practice at commercial farms to prove their effectiveness. Future research should include controlled feeding trials where dietary interventions are guided by microbiome monitoring to evaluate their effects on growth performance, feed efficiency, and disease resistance under defined experimental conditions. In addition, there is a need to develop and validate next-generation microbiome-based interventions, including defined microbial consortia, synbiotics, postbiotics, and microbial metabolites, to achieve consistent and reproducible benefits across diverse production systems.

## 9. Conclusions

The evidence now available very clearly shows that the gut microbiome plays a critical role in broiler health and productivity, such as in nutrient digestion, production of microbial metabolites, regulation of the immune system, intestinal barrier function, and disease resistance. An extensive body of research has also demonstrated that dietary changes, probiotics, prebiotics and other antibiotic alternatives can impact microbial structure and function, which in turn can impact growth performance and feed efficiency. In addition, environmental and management influences, including housing, feed management and stress exposure, have been shown to be key factors in the structure and stability of the microbiome. In spite of these developments, the mechanisms of how the microbiome influences the highly variable responses to microbiome-targeted interventions are not well understood. These inconsistencies have been increasingly attributed to the interactions between complex microbial communities, host physiology, genetic makeup, nutrition, and environment. In this review, a new conceptual model of the interaction between the microbiome and host and environment is proposed, which will help to better understand these interactions and their combined effect on broiler productivity. This systems-level view offers optimistic roadmap for some of the future poultry production systems, but there are some aspects with which we are only beginning to hypothesize. Specifically, techniques of multi-omics technologies, predictive microbiome modelling, artificial intelligence and precision nutrition for real-time micro-biome management are in a nascent stage of development. These methods should be tested under commercial production conditions in the future and robust biomarkers should be identified that can predict the functionality of the microbiome and the production outcomes. In summary, the combination of microbiome science and host physiology and environmental management presents a strong potential to decrease dependence on antibiotics and enhance the sustainability of poultry production. However, shifting from descriptive microbiome studies to mechanistic, predictive, and systems-based approaches that enable the translation of microbiome knowledge to consistent on-farm outcomes will be a challenge to achieve.

## Data Availability

No new data were generated for this review article. Data sharing is not applicable to this article.
